# Nocturnal Pain in Patients with Knee Osteoarthritis: A Single-Center Cross-Sectional Study

**DOI:** 10.3390/healthcare14172823

**Published:** 2026-09-03

**Authors:** Köksal Sarıhan, Erdem Özkaya, Cemil Güner, Ali Yazıcı, Osman Topçu, Ceyhun Yurtsever, Erhan Özdemir

**Affiliations:** 1 Maçka Ömer Burhanoğlu Physical Therapy and Rehabilitation Hospital, Trabzon 61750, Türkiye; 2 Department of Family Medicine, Kanuni Training and Research Hospital, Trabzon 61220, Türkiye

**Keywords:** knee osteoarthritis, pain, nocturnal pain, neuropathic pain, central sensitization, sleep quality

## Abstract

**Highlights:**

**What are the main findings?**

- We found that the prevalence of nocturnal pain (NP) was 64.7% in our knee osteoarthritis (KOA) patient group.
- We found that KOA disease severity was higher in the NP group.
- In the secondary unadjusted comparisons, neuropathic pain, anxiety, irritable bowel syndrome, and fibromyalgia were more common in the NP group; the NP group had higher central sensitivity scores, worse sleep quality scores, and a higher number of hospital visits.

**What are the implications of the main findings?**

- Given that it affects more than half of patients with KOA and is associated with higher disease severity, we emphasize the need for careful management of NP in KOA patients.

**Abstract:**

**Objectives**: The aim of this study was to determine the differences between groups with and without nocturnal pain (NP) in patients with knee osteoarthritis (KOA) in terms of radiographic stage, disease severity, neuropathic pain, central sensitivity, sleep quality, physical activity level, and number of hospital visits. **Methods**: The study included 150 patients with KOA (101 women, 49 men; mean age 64 years (IQR: 9; range: 40–81)). Kellgren–Lawrence (KL) grading was used for radiographic staging, the Western Ontario and McMaster Universities Osteoarthritis Index (WOMAC) for KOA disease severity, the Douleur Neuropathique 4 (DN-4) questionnaire for neuropathic pain, the Central Sensitization Inventory (CSI) for central sensitization, the Pittsburgh Sleep Quality Index (PSQI) for sleep quality, and the International Physical Activity Questionnaire–Short Form (IPAQ-SF) for physical activity level. **Results**: In our KOA patient group, the prevalence of NP was found to be 64.7%. A multivariable binary logistic regression analysis was performed as the primary inferential analysis. Age, sex, educational level, body mass index, disease duration, KL grade, and WOMAC total score were entered simultaneously into the model. After adjustment for the included covariates, only the WOMAC total score remained independently associated with NP. Each one-point increase in the WOMAC total score was associated with an 11.7% increase in the odds of NP (adjusted OR = 1.117, 95% CI 1.068–1.168, *p* < 0.001). **Conclusions**: Given that it affects more than half of patients with KOA and is associated with higher disease severity, we emphasize the need for careful management of NP in KOA patients.

## 1. Introduction

Osteoarthritis (OA) is the most common joint disease, with its prevalence increasing with age. In one study, the prevalence of radiographic knee osteoarthritis (KOA) was found to be 19.2% among participants older than 45 years and 43.7% among individuals older than 80 years [1]. KOA constitutes an important socioeconomic burden in societies by causing chronic pain and impairing physical independence [2]. The development of OA is influenced by genetic, biological, biochemical, and mechanical factors. In KOA, conservative treatment options are initially applied. If the patient’s symptoms do not improve after conservative treatments, surgical treatment options may be offered. Many new treatment methods, including virtual reality, are also being investigated for patients with KOA [3].

Pain is typically the initial symptom of KOA [2,4]. Nociceptive signals in OA arise from free nerve endings within the synovium, periosteum, and tendons [5,6]. KOA pain typically begins with a mechanical character and may eventually progress to pain at rest or continuous pain [7]. Both peripheral and central nociceptive and neuropathic pathways contribute to the complex pain experience of OA [5,8,9]. Neuropathic pain frequently presents in OA patients [9,10]. Consequently, pain associated with OA emerges as a combination of mechanical, biological, social, and psychological processes [8,11,12,13].

Nocturnal pain (NP) is an important component of the pain experience in KOA and is reported as a major concern in these patients [7]. The mechanisms related to NP in patients with KOA have not been clearly established. In addition to excessive mechanical load and synovitis resulting from daytime activities, neuropathic pain, central sensitization, psychological disorders, and poor sleep quality have also been associated with NP [10,14,15,16]. Conventional medical therapy does not adequately relieve joint pain in some patients, and symptoms may continue even after joint replacement. This persistence suggests that peripheral and central sensitivity may be related to OA pain [17]. Central sensitization is considered one of the key factors in identifying chronic pain in patients with KOA [15,16]. The International Association for the Study of Pain describes central sensitization as an enhanced response of central nervous system nociceptors to normal, harmless, or below-threshold afferent stimuli [18]. Sleep problems frequently occur among people with OA [19]. Although the cause of sleep disturbance in OA is unclear, pain is likely a major contributing factor. At the same time, impaired sleep is known to have negative effects on pain and psychological status [11,19].

Studies show that NP differs from daytime pain and is generally described as more severe [7,11]. NP increases dissatisfaction and reduces quality of life in patients with KOA [15]. Disturbing NP is an important determining factor for physicians when deciding whether a patient should undergo total joint replacement [20]. Accurate understanding, management, and control of NP are critically important. Having more information about NP will also be beneficial for physicians in informing patients. NP in KOA has been investigated in many recent scientific studies [7,9,15]. However, there are still unclear issues regarding KOA. Previous studies have examined the relationship between radiographic staging and many clinical features of KOA, but the relationship between NP and radiographic staging has not been clearly established [15,21,22]. There are few studies on the role of central sensitization and neuropathic pain in NP among patients with KOA [7,9,15,23]. Evidence concerning the association between NP and physical activity in KOA is likewise limited [7,11]. Moreover, as far as we are aware, the literature has not compared the number of hospital visits made by KOA patients with versus without NP. The aim of this study was to determine the differences between groups with and without NP in patients with KOA in terms of radiographic stage, disease severity, neuropathic pain, central sensitivity, sleep quality, physical activity level, and number of hospital visits.

## 2. Materials and Methods

### 2.1. Study Design

Participant recruitment for this cross-sectional study was carried out at the physical medicine and rehabilitation outpatient clinic of a district state hospital in Turkey between 11 June 2023 and 11 September 2023. Participants were included in the study consecutively.

### 2.2. Participants

The study included 150 consecutive participants older than 40 years who were followed by a physical medicine and rehabilitation specialist with a diagnosis of KOA and who had a Kellgren–Lawrence (KL) radiographic stage of 2 or higher (101 women, 49 men; median age 64 years (IQR: 9; range: 40–81)). Patients with total knee arthroplasty or arthrodesis, inflammatory rheumatic disease, severe psychiatric disorder, a history of cancer, and emergency patients were excluded from the study. One hundred eighty-six participants who met the inclusion criteria were screened for participation in the study during the specified period. Thirty-six individuals were excluded due to exclusion criteria. No one declined to participate in the study. We did not experience any data loss for any of the participants.

### 2.3. Study Ethics

Written informed consent was obtained from all participants. We conducted the study in accordance with the Declaration of Helsinki. The university hospital ethics committee approved the study on 1 June 2023 (Decision No: B.30.2.ATA.0.01.00/457).

### 2.4. Outcome Measures

First, demographic data of the participants, including age, gender, and educational status, were recorded for the study. Subsequently, comorbid diseases, disease duration, number of hospital visits in the last year, and presence of NP were noted. Body mass index (BMI) was calculated by measuring height and weight. The presence of NP was determined using the question, “Do you have pain at night?” [24,25]. This question assessed whether the participants had experienced any nocturnal knee pain in the past 48 h [24,25]. Weight-bearing anteroposterior knee radiographs were evaluated, and KL grading was performed. Finally, the Western Ontario and McMaster Universities Osteoarthritis Index (WOMAC) was used to assess KOA disease severity, the Douleur Neuropathique 4 (DN-4) questionnaire was used to detect the presence of neuropathic pain, the Central Sensitization Inventory (CSI) was used to assess central sensitization, the Pittsburgh Sleep Quality Index (PSQI) was used to assess sleep quality, and the International Physical Activity Questionnaire–Short Form (IPAQ-SF) was used to assess physical activity status. Participants were asked to answer the questions based on their most symptomatic knees. The questionnaires were completed by the participants. Researchers provided assistance to participants if they requested it.

KL grading was used for the radiographic grading of KOA [26]. The most symptomatic knees of the participants were included in the KL grading. According to the KL scale, a normal knee radiograph was classified as grade 0; the presence of doubtful osteophytes with a normal joint space as grade 1; definite osteophytes with doubtful joint space narrowing as grade 2; moderate osteophytes with moderate joint space narrowing as grade 3; and large osteophytes with severe joint space narrowing as grade 4. KL grading of all participants was performed by the same physical medicine and rehabilitation specialist (researcher A.Y.). The researcher responsible for KL grading did not have access to the patients’ other clinical data during the grading process.

The WOMAC assesses pain, stiffness, and physical function in patients with KOA over the previous 48 h [24]. The scale consists of 24 items and three subdimensions. It is a five-point Likert-type scale (0 = none, 1 = mild, 2 = moderate, 3 = severe, 4 = extreme). Pain is assessed in the first section of the scale (5 questions), morning stiffness in the second section (2 questions), and functional disability in the third section (17 questions). Possible scores are 0–20 for pain, 0–8 for stiffness, and 0–68 for physical function. Greater values reflect more severe pain, stiffness, and functional impairment. Tüzün et al. established the validity and reliability of the Turkish version [25].

The DN-4 questionnaire was used to determine the presence of neuropathic pain. This scale consists of 10 questions, 7 of which assess symptoms and 3 of which are determined by clinical examination. The symptoms assessed are burning sensation, painful cold sensation, electric shocks, tingling, pins and needles, numbness, and itching. The examination findings are hypoesthesia to light touch, hypoesthesia to pinprick, and brushing allodynia. Each question answered “yes” is scored as 1 point. To calculate the total score, the scores obtained from symptom assessment and clinical examination are summed. The maximum total score is 10, and neuropathic pain is considered to be present in patients scoring 4 or higher [27]. Çevik et al. adapted the DN-4 into Turkish and established its validity and reliability [28].

The CSI consists of two sections: Part A, which evaluates symptoms thought to be associated with central sensitization syndromes; and Part B, which rapidly assesses whether the patient has previously received a specific diagnosis that may be associated with central sensitization. Part A contains 25 items on symptom frequency and yields a score from 0 to 100. Each item is scored from 0 to 4: 0 for never, 1 for rarely, 2 for sometimes, 3 for often, and 4 for always. Higher CSI scores represent a greater burden of symptoms linked to central sensitization. Part B records prior physician diagnoses of conditions classified as central sensitization syndromes. A score of 40 or higher indicates acceptable sensitivity and specificity in accurately identifying patients with central sensitivity syndrome [29]. Central sensitization levels can be divided into 5 different groups based on the total score obtained from the scale. These are: subclinical level (0–29 points), mild level (30–39 points), moderate level (40–49 points), severe level (50–59 points), and very severe level (60 and above points) [30]. The inventory is a screening measure with established sensitivity and specificity in chronic pain populations [31]. Düzce Keleş et al. demonstrated the validity and reliability of its Turkish form [32].

The PSQI assesses sleep quality during the preceding month through 19 questions [33]. Its seven domains are subjective sleep quality, sleep latency, sleep duration, sleep efficiency, sleep disturbances, sleep-medication use, and daytime dysfunction. The global score varies from 0 to 21, with values of 5 or above indicating poor sleep quality. Ağargün et al. validated the Turkish version and confirmed its reliability [34].

The IPAQ-SF was used to determine individuals’ physical activity level [35]. This seven-question questionnaire evaluates in detail the amount of walking performed during the previous week and the amount of moderate and vigorous physical activity performed in work, transportation, housework, gardening, and leisure-time activities. For data analysis, walking was evaluated as 3.3 metabolic equivalents of task (METs), moderate-intensity physical activity as 4.0 METs, and vigorous physical activity as 8.0 METs, and calculations were performed accordingly. For each domain, minutes, days, and MET value were multiplied separately to obtain “MET-minutes/week.” These values were summed to calculate the total value for that week. Consequently, physical activity level was divided into three groups: 0–599 MET-minutes/week: inactive (Category 1), 600–2999 MET-minutes/week: minimally active (Category 2), and >3000 MET-minutes/week: very active (Category 3). The validity and reliability study of the questionnaire in Türkiye was conducted by Öztürk M et al. [36].

### 2.5. Statistical Analysis

Statistical analyses were performed using IBM SPSS Statistics for Windows, version 23.0 (IBM Corp., Armonk, NY, USA). The distributions of numerical variables were evaluated using skewness and kurtosis coefficients. Values between −1.5 and +1.5 were considered indicative of an approximately normal distribution. Normally distributed numerical variables were presented as mean ± standard deviation, whereas non-normally distributed variables were presented as median [interquartile range (IQR)]. Categorical variables were summarized as frequencies and percentages.

Age, BMI, disease duration, KL grade, CSI Part A score, PSQI score, and number of hospital visits were compared between participants with and without NP using the Mann–Whitney U test. KL grade was treated as an ordinal variable. WOMAC pain and stiffness scores were compared using the independent-samples t-test. Homogeneity of variance was assessed using Levene’s test. Because the homogeneity-of-variance assumption was not satisfied for WOMAC physical function and total scores, these variables were compared using Welch’s *t*-test.

Associations between categorical variables and NP were evaluated using Pearson’s chi-square test. Fisher’s exact test was used when any expected cell frequency was less than 5.

Effect sizes and 95% confidence intervals were reported for the group comparisons. Odds ratios were calculated for binary categorical variables. Because no cases of fibromyalgia were observed in the group without NP, the fibromyalgia comparison was summarized using the absolute prevalence difference and its 95% confidence interval. Prevalence differences were also used for other sparse comparisons containing a zero cell. Cramér’s V was reported for the three category IPAQ-SF comparison. Hedges’ g was used for normally distributed numerical variables, whereas rank-biserial correlation was used for non-normally distributed or ordinal variables. Ninety-five percent confidence intervals for Hedges’ g and rank-biserial correlations were estimated using 5000 percentile bootstrap resamples.

A multivariable binary logistic regression analysis was performed to identify factors independently associated with NP. The presence of NP was entered as the dependent variable (no = 0, yes = 1). Age, sex, educational level, BMI, disease duration, KL grade, and WOMAC total score were selected by the research team as clinically relevant covariates and entered simultaneously into the model. Female sex and primary-school education or below were used as the reference categories. KL grade was entered as an ordinal covariate, and its odds ratio represented the change associated with a one-grade increase. Adjusted odds ratios, 95% confidence intervals, and *p* values were reported.

Multicollinearity was assessed using variance inflation factor and tolerance values, with VIF values below 5 and tolerance values above 0.20 considered acceptable. Overall model significance was evaluated using the omnibus likelihood-ratio chi-square test. Model calibration was assessed using the Hosmer–Lemeshow goodness-of-fit test. Cox–Snell and Nagelkerke pseudo-R^2^ values, overall classification accuracy, sensitivity, and specificity were also reported using a predicted-probability cut-off of 0.50.

The multivariable logistic regression was considered the primary inferential analysis. Bivariate group comparisons were considered secondary and exploratory. Because these comparisons included several clinically correlated variables and were intended to characterize the groups rather than test independent confirmatory hypotheses, no formal adjustment for multiple comparisons was applied. Accordingly, *p* values from the bivariate analyses should be considered nominal and interpreted cautiously. All statistical tests were two-sided, and *p* < 0.05 was considered statistically significant.

## 3. Results

A total of 150 participants with KOA were included in the analysis. NP was reported by 97 participants, corresponding to a prevalence of 64.7% (95% CI 56.7–71.9%). Fifty-three participants (35.3%) did not report NP.

In the secondary unadjusted comparisons, the proportion of women was higher in the NP group than in the group without NP (79.4% vs. 45.3%, *p* < 0.001; OR = 4.65, 95% CI 2.24–9.66). Primary-school education or below was also more frequent among participants with NP (78.4% vs. 56.6%, *p* = 0.005; OR = 2.77, 95% CI 1.34–5.74).

Neuropathic pain was more frequent in the NP group (16.5% vs. 3.8%, *p* = 0.022; OR = 5.04, 95% CI 1.11–22.83). Fibromyalgia was present in 26.8% of participants with NP but was not reported in the group without NP (prevalence difference = 26.8 percentage points, 95% CI 16.5–36.4; *p* < 0.001). Irritable bowel syndrome (23.7% vs. 9.4%, *p* = 0.032; OR = 2.98, 95% CI 1.06–8.38) and anxiety (19.6% vs. 3.8%, *p* = 0.008; OR = 6.21, 95% CI 1.39–27.82) were also more frequent. These estimates, particularly those with wide confidence intervals, should be interpreted cautiously.

No statistically significant differences were observed between the groups in hypertension, diabetes mellitus, restless legs syndrome, chronic fatigue syndrome, migraine or tension-type headache, depression, or physical-activity category. Temporomandibular joint disorder, multiple chemical sensitivity, and whiplash injury were not reported in either group; therefore, effect sizes could not be estimated for these variables (Table 1).

Age, BMI, and disease duration did not differ significantly between participants with and without NP. KL grade was higher in the NP group than in the group without NP (median [IQR]: 3 [1] vs. 2 [1]; *p* = 0.008; rank-biserial correlation = 0.234, 95% CI 0.072–0.383).

Participants with NP had higher CSI Part A scores than those without NP (22 [14] vs. 8 [9]; *p* < 0.001; rank-biserial correlation = 0.664, 95% CI 0.519–0.798). PSQI scores were also higher (8 [6] vs. 4 [4]; *p* < 0.001; rank-biserial correlation = 0.574, 95% CI 0.416–0.715). Overall, 94 of the 150 participants had a PSQI score of at least 5, corresponding to a poor-sleep-quality prevalence of 62.7%.

The number of hospital visits during the preceding year was higher among participants with NP (2 [2] vs. 1 [1]; *p* = 0.001; rank-biserial correlation = 0.304, 95% CI 0.133–0.467). These numerical and ordinal comparisons were unadjusted and exploratory (Table 2).

All WOMAC scores were higher among participants with NP in the unadjusted analyses. The mean WOMAC pain score was 9.42 ± 2.71 in the NP group and 5.30 ± 2.37 in the group without NP (*p* < 0.001; Hedges’ g = 1.580, 95% CI 1.223–1.991). The corresponding WOMAC stiffness scores were 1.85 ± 1.76 and 1.13 ± 1.62 (*p* = 0.016; Hedges’ g = 0.414, 95% CI 0.083–0.758).

Participants with NP also had higher WOMAC physical-function scores (30.92 ± 7.00 vs. 20.28 ± 8.70; *p* < 0.001; Hedges’ g = 1.385, 95% CI 1.006–1.821) and WOMAC total scores (43.78 ± 10.14 vs. 28.25 ± 12.37; *p* < 0.001; Hedges’ g = 1.407, 95% CI 1.040–1.857). The effect sizes for WOMAC pain, physical function, and total score indicated large unadjusted between-group differences (Table 3).

A multivariable binary logistic regression analysis was performed as the primary inferential analysis (Table 4). Age, sex, educational level, BMI, disease duration, KL grade, and WOMAC total score were entered simultaneously into the model. The overall model was statistically significant (χ^2^[7] = 57.281, *p* < 0.001) and yielded a Cox–Snell R^2^ of 0.317 and a Nagelkerke R^2^ of 0.436. The model demonstrated acceptable calibration according to the Hosmer–Lemeshow test (χ^2^[8] = 6.653, *p* = 0.575). The overall classification accuracy was 76.0%, with a sensitivity of 86.6% and a specificity of 56.6%. No problematic multicollinearity was detected (VIF range 1.105–1.783; tolerance range 0.561–0.905).

After adjustment for the included covariates, only the WOMAC total score remained independently associated with NP. Each one-point increase in the WOMAC total score was associated with an 11.7% increase in the odds of NP (adjusted OR = 1.117, 95% CI 1.068–1.168, *p* < 0.001). Age (adjusted OR = 1.035, 95% CI 0.963–1.112, *p* = 0.350), male sex (adjusted OR = 0.598, 95% CI 0.198–1.807, *p* = 0.362), educational level (adjusted OR = 0.899, 95% CI 0.332–2.434, *p* = 0.834), BMI (adjusted OR = 0.943, 95% CI 0.847–1.050, *p* = 0.283), disease duration (adjusted OR = 1.013, 95% CI 0.902–1.137, *p* = 0.833), and KL grade (adjusted OR = 1.299, 95% CI 0.644–2.617, *p* = 0.465) were not independently associated with NP.

Thus, although several demographic, radiographic, and clinical characteristics differed between the groups in the secondary unadjusted analyses, only greater disease severity, as reflected by the WOMAC total score, remained independently associated with NP in the adjusted model.

## 4. Discussion

We found that the prevalence of NP was 64.7% in our KOA patient group. We found that disease severity, as assessed by WOMAC, was higher in the NP group. Neuropathic pain, central sensitization, anxiety, irritable bowel syndrome, and fibromyalgia were more common. We also found that the NP group had worse sleep quality scale scores and a higher number of hospital visits.

Pain is the main symptom of KOA [2,8]. Previous studies have reported a wide range for the prevalence of NP in patients with KOA (14–85%) [7,11,15]. The reasons for these differences between studies may include personal characteristics related to pain perception, demographic differences such as age, sex, and educational status, differences in disease severity and radiographic grades, and exclusion criteria. Consistent with previous findings, our study found that the prevalence of NP in our KOA patient group was 64.7%. It appears that more than half of patients with KOA experience NP.

In a study conducted in patients with KOA, NP was shown to increase with the radiographic severity of the disease [21]. In another study, patients with KOA stated that NP became more intense as their disease progressed [11]. In our regression analysis, unlike what has been reported in the literature, we did not find a difference in KL grade between the groups with and without NP. This may be because we assessed NP with only one question and did not use a clinical scale to measure pain. This issue should be investigated in studies with larger sample sizes. According to our study data, unlike KL grading, disease severity assessed with WOMAC was worse in the NP group. In the regression analysis, disease severity, as assessed by WOMAC, was also worse. In KOA patients, NP is associated with higher disease severity scores.

Pain in KOA patients does not arise solely from mechanical and structural causes [12,13]. Supporting this information, neuropathic pain was more common in the NP group than in the without NP group in our study. In addition, according to the data of our study, the NP group had more frequent hospital visits. Our study data demonstrate the importance of NP in this respect, as it may cause an increased hospital burden. These analyses were intended to characterize the groups and were considered exploratory. Therefore, these findings should be interpreted cautiously and confirmed by studies with sufficient power.

Fingleton et al. [37] reported that central sensitization could contribute to KOA-related pain. In one study, an association was found between NP and central sensitization in KOA patients [15]. Consistent with prior findings, in our study, we found that the CSI score was higher in the NP group. Furthermore, in our study, we found that the incidence of fibromyalgia and irritable bowel syndrome, both of which are central sensitivity syndromes, was higher. We additionally assessed anxiety and depression through CSI Part B. Psychological factors, including anxiety and depression, alter pain perception in OA through various pathways [38]. One study reported a significant correlation between joint pain and depressive status [39]. Unlike previous studies, our study found no difference in the frequency of depression diagnoses between the patients with and without NP. This may be because we addressed this issue only with CSI Part B and did not investigate depressive status using other clinical diagnostic tools. In line with the literature, the prevalence of anxiety was higher in the NP group in our study. According to one study, the onset and increasing severity of NP lead to concerns about the future in patients with KOA [11]. In the same study, participants with knee pain stated that during the night, they were more aware of and concerned about their NP and discomfort because of mental distractions and lack of stimulation. In conclusion, we found higher central sensitization scores and anxiety rates in the NP group. However, these analyses aimed to characterize the groups and are exploratory in nature. These findings should be interpreted with caution.

In one study, 63.5% of KOA patients were found to experience sleep problems [19]. In another study, the rate of patients with KOA who had poor sleep quality was 81.1% [40]. In line with the literature, in our study, 62.7% of KOA patients had poor sleep quality. In KOA patients, NP has been shown to be the primary cause of sleep disturbances [9]. According to one study, NP significantly impairs sleep quality and leads to a decrease in quality of life in patients with KOA [21]. Supporting the literature, according to the data of our study, sleep quality scores were also poorer in the NP group. While these findings suggest that KOA patients with NP have lower sleep quality scores, these data should be interpreted with caution due to the exploratory nature of the study.

In one study, KOA patients stated that they could predict the occurrence or severity of NP because it depended on the level of activity they engaged in during the day [11]. Although participants could predict NP, they stated that this situation often did not prevent them from being active during the day and that they showed a degree of acceptance. Supporting this view, in our study, we did not detect a difference in physical activity status between KOA patients with and without NP. One reason for this may be that patients with KOA can tolerate NP to a certain extent and have accepted it. Another reason may be that, in line with physicians’ recommendations, they were encouraged to remain as physically active as possible to the extent permitted by their pain. Another reason might be that, due to the cross-sectional design, we were unable to determine the temporal relationships between NP and physical activity. Additionally, it should be considered that the IPAQ-SF has limited sensitivity and may not be the most suitable method for assessing physical activity in older adults with joint disease.

This study has several limitations. First, this study had a single-center design. Experiential pain may be affected by geographical and personal factors. Second, the cross-sectional design prevents determination of temporal sequence or causality. Third, nocturnal knee pain was assessed with a single question and not measured using a pain scale. This could lead to patients experiencing mild NP being grouped similarly to those experiencing severe nocturnal pain, potentially masking significant differences and relationships. Fourth, seasonal variation in pain reporting was not considered in our study. Pain perception and activity levels may vary between seasons. Fifth, central-sensitization-related symptoms were evaluated using the self-reported Central Sensitization Inventory. Although the CSI is a validated screening instrument, it does not provide a definitive clinical or physiological diagnosis of central sensitization. Moreover, fibromyalgia, anxiety, depression, irritable bowel syndrome, and other central-sensitivity-related conditions were evaluated using the diagnostic history recorded in CSI Part B rather than condition-specific validated instruments or standardized clinical diagnostic assessments. Sixth, potentially relevant clinical factors—including analgesic and sleep-medication use, recent physical therapy, previous intra-articular injections, and other pain-related treatments—were not recorded. Seventh, psychological factors that may affect pain perception, such as pain catastrophizing, fear avoidance, and pain self-efficacy, were also not assessed. Eighth, the sample size was not determined specifically for the multivariable logistic regression analysis. Although the final seven-predictor model converged, demonstrated acceptable calibration, and showed no evidence of problematic multicollinearity, the number of participants—particularly the 53 participants without nocturnal pain—limited the complexity and precision of the adjusted analysis. The model also had a specificity of 56.6%, indicating limited ability to correctly classify participants without nocturnal pain. The model’s moderate specificity suggests that factors not captured in our model may also contribute to NP, highlighting the need for further research. To limit overparameterization, the multivariable model was restricted to age, sex, educational level, BMI, disease duration, KL grade, and WOMAC total score. Consequently, the independent contributions of CSI Part A score, PSQI score, neuropathic pain, fibromyalgia, anxiety, and number of hospital visits were not evaluated in the final adjusted model. Differences in these variables were based on secondary unadjusted analyses and should not be interpreted as evidence of independent associations with nocturnal pain. Ninth, numerous secondary bivariate comparisons were performed without a formal adjustment for multiple testing. These analyses were intended to characterize the groups and were considered exploratory; however, the absence of a multiplicity correction increases the possibility of type I errors. Although effect sizes and 95% confidence intervals were reported to reduce reliance on *p* values alone, some nominally significant findings may have occurred by chance. The secondary findings should therefore be interpreted cautiously and confirmed in adequately powered prospective multicenter studies using quantitative and multidimensional measures of nocturnal pain. Finally, although all eligible patients presenting during the predefined recruitment period were consecutively included, the absence of a valid a priori sample size calculation should be considered when interpreting the statistical precision of the findings, particularly for comparisons involving less frequent outcomes.

## 5. Conclusions

We found that NP is common in KOA patients and that disease severity is higher in the NP group. In the secondary unadjusted comparisons, neuropathic pain, anxiety, irritable bowel syndrome, and fibromyalgia were more common in the NP group; the NP group had higher central sensitivity scores, worse sleep quality scores, and a higher number of hospital visits. Given that it affects more than half of patients with KOA and is associated with higher disease severity, we emphasize the need for careful management of NP in KOA patients. Future research should examine how NP relates to cognitive function in KOA and evaluate potential therapeutic approaches.

## Figures and Tables

**Table 1:** Comparison of the Demographic Data and Clinical Characteristics of the Participants With and Without NP (*n* = 150).

Variable	Total	Without NP (*n* = 53)	With NP (*n* = 97)	*p* Value	Effect Size (95% CI)
**Sex**				<0.001	OR = 4.65 (2.24–9.66)
Female	101 (67.3)	24 (45.3)	77 (79.4)		
Male	49 (32.7)	29 (54.7)	20 (20.6)		
**Educational status**				0.005	OR = 2.77 (1.34–5.74)
Primary school or below	106 (70.7)	30 (56.6)	76 (78.4)		
Secondary school or higher	44 (29.3)	23 (43.4)	21 (21.6)		
**Hypertension**				0.077	OR = 1.84 (0.93–3.63)
No	62 (41.3)	27 (50.9)	35 (36.1)		
Yes	88 (58.7)	26 (49.1)	62 (63.9)		
**Diabetes mellitus**				0.407	OR = 1.46 (0.59–3.59)
No	122 (81.3)	45 (84.9)	77 (79.4)		
Yes	28 (18.7)	8 (15.1)	20 (20.6)		
**Neuropathic pain**				0.022	OR = 5.04 (1.11–22.83)
No	132 (88.0)	51 (96.2)	81 (83.5)		
Yes	18 (12.0)	2 (3.8)	16 (16.5)		
**Restless legs** **syndrome**				0.540	PD = 2.1% (−4.9–7.2%)
No	148 (98.7)	53 (100)	95 (97.9)		
Yes	2 (1.3)	0 (0)	2 (2.1)		
**Chronic fatigue** **syndrome**				1.000	PD = 1.0% (−5.8–5.6%)
No	149 (99.3)	53 (100)	96 (99.0)		
Yes	1 (0.7)	0 (0)	1 (1.0)		
**Fibromyalgia**				<0.001	PD = 26.8% (16.5–36.4%)
No	124 (82.7)	53 (100)	71 (73.2)		
Yes	26 (17.3)	0 (0)	26 (26.8)		
**Temporomandibular joint disorder**				—	Not estimable
No	150 (100)	53 (100)	97 (100)		
Yes	0 (0)	0 (0)	0 (0)		
**Migraine/tension-type** **headache**				1.000	OR = 1.66 (0.17–16.36)
No	146 (97.3)	52 (98.1)	94 (96.9)		
Yes	4 (2.7)	1 (1.9)	3 (3.1)		
**Irritable bowel** **syndrome**				0.032	OR = 2.98 (1.06–8.38)
No	122 (81.3)	48 (90.6)	74 (76.3)		
Yes	28 (18.7)	5 (9.4)	23 (23.7)		
**Multiple chemical** **sensitivity**				—	Not estimable
No	150 (100)	53 (100)	97 (100)		
Yes	0 (0)	0 (0)	0 (0)		
**Whiplash injury**				—	Not estimable
No	150 (100)	53 (100)	97 (100)		
Yes	0 (0)	0 (0)	0 (0)		
**Anxiety**				0.008	OR = 6.21 (1.39–27.82)
No	129 (86.0)	51 (96.2)	78 (80.4)		
Yes	21 (14.0)	2 (3.8)	19 (19.6)		
**Depression**				0.379	OR = 1.62 (0.55–4.77)
No	131 (87.3)	48 (90.6)	83 (85.6)		
Yes	19 (12.7)	5 (9.4)	14 (14.4)		
**IPAQ-SF category**				0.135	Cramér’s V = 0.163
Category 1	33 (22.0)	9 (17.0)	24 (24.7)		
Category 2	55 (36.7)	25 (47.2)	30 (30.9)		
Category 3	62 (41.3)	19 (35.8)	43 (44.3)		

Data are presented as *n* (%). Comparisons were performed using Pearson’s chi-square test or Fisher’s exact test when expected cell frequencies were less than 5. Effect sizes for binary categorical comparisons are presented as odds ratios (ORs) with 95% confidence intervals (CIs). ORs represent the odds of nocturnal pain (NP) in the first listed category for sex and educational status and in the “Yes” category for clinical conditions, relative to their respective reference categories. For sparse comparisons containing a zero cell, prevalence differences (PDs; prevalence in the NP group minus prevalence in the group without NP) with 95% CIs are reported. Cramér’s V is reported for the three-category IPAQ-SF comparison. Effect sizes could not be estimated for variables with no observed cases in either group. NP: cocturnal pain; IPAQ-SF: International Physical Activity Questionnaire–Short Form.

**Table 2:** Demographic Characteristics of Participants, Scale Scores, and Comparison of Data Between Participants with and without NP (*n*:150).

Variable	Total (*n* = 150)	Without NP (*n* = 53)	With NP (*n* = 97)	*p* Value	Rank-Biserial Correlation (95% CI)
**Age, years**	64 [9]	64 [9]	64 [10]	0.470	0.072 (−0.113–0.261)
**BMI, kg/m^2^**	30.2 [6.4]	29.0 [5.2]	30.5 [6.7]	0.105	0.161 (−0.039–0.357)
**Disease duration, years**	2 [4]	2 [4]	3 [4]	0.070	0.173 (−0.013–0.347)
**KL grade**	2 [1]	2 [1]	3 [1]	0.008	0.234 (0.072–0.383)
**CSI Part A score**	17 [18]	8 [9]	22 [14]	<0.001	0.664 (0.519–0.798)
**PSQI total score**	6 [6]	4 [4]	8 [6]	<0.001	0.574 (0.416–0.715)
**Number of hospital visits**	2 [2]	1 [1]	2 [2]	0.001	0.304 (0.133–0.467)

Data are presented as median [interquartile range]. Between-group comparisons were performed using the Mann–Whitney U test. KL grade was treated as an ordinal variable. Effect sizes are presented as rank-biserial correlations with 95% bootstrap confidence intervals based on 5000 resamples. Positive effect-size values indicate higher ranks in the nocturnal pain (NP) group. Reported *p* values are nominal and should be interpreted as exploratory. NP: nocturnal pain; BMI: body mass index; KL: Kellgren–Lawrence; CSI: Central Sensitization Inventory; PSQI: Pittsburgh Sleep Quality Index; CI: confidence interval.

**Table 3:** Comparison of KL Grades and WOMAC Scores of Participants With and Without NP (*n* = 150).

Variable	Total (*n* = 150)	Without NP (*n* = 53)	With NP (*n* = 97)	*p* Value	Hedges’ g (95% CI)
**WOMAC pain**	7.97 ± 3.26	5.30 ± 2.37	9.42 ± 2.71	<0.001	1.580 (1.223–1.991)
**WOMAC stiffness**	1.59 ± 1.74	1.13 ± 1.62	1.85 ± 1.76	0.016	0.414 (0.083–0.758)
**WOMAC physical function**	27.16 ± 9.16	20.28 ± 8.70	30.92 ± 7.00	<0.001	1.385 (1.006–1.821)
**WOMAC total score**	38.29 ± 13.23	28.25 ± 12.37	43.78 ± 10.14	<0.001	1.407 (1.040–1.857)

Data are presented as mean ± standard deviation. WOMAC pain and stiffness scores were compared using the independent-samples t-test. Because the homogeneity-of-variance assumption was not satisfied for WOMAC physical-function and total scores, these variables were compared using Welch’s t-test. Effect sizes are presented as Hedges’ g with 95% bootstrap confidence intervals based on 5000 resamples. Positive effect-size values indicate higher scores in the nocturnal pain (NP) group. Reported *p* values are nominal and should be interpreted as exploratory. NP: nocturnal pain; WOMAC: Western Ontario and McMaster Universities Osteoarthritis Index; CI: confidence interval.

**Table 4:** Multivariable binary logistic regression analysis of factors associated with NP (*n* = 150).

Variable	B	SE	Wald χ^2^	Adjusted OR	95% CI	*p* Value
**Age, years**	0.034	0.037	0.872	1.035	0.963–1.112	0.350
**Male sex**	−0.514	0.564	0.831	0.598	0.198–1.807	0.362
**Secondary school or higher education**	−0.107	0.508	0.044	0.899	0.332–2.434	0.834
**BMI, kg/m^2^**	−0.059	0.055	1.155	0.943	0.847–1.050	0.283
**Disease duration, years**	0.012	0.059	0.044	1.013	0.902–1.137	0.833
**KL grade**	0.261	0.358	0.534	1.299	0.644–2.617	0.465
**WOMAC total score**	0.110	0.023	23.095	1.117	1.068–1.168	<0.001

Model characteristics: Omnibus χ^2^(7) = 57.281, *p* < 0.001; −2 log likelihood = 137.565; Cox–Snell R^2^ = 0.317; Nagelkerke R^2^ = 0.436; Hosmer–Lemeshow χ^2^(8) = 6.653, *p* = 0.575. Overall classification accuracy = 76.0%; sensitivity = 86.6%; specificity = 56.6%. The dependent variable was the presence of nocturnal pain (NP) (no = 0, yes = 1). All predictors were entered simultaneously. Reference categories were female sex and primary-school education or below. Adjusted ORs for continuous and ordinal variables represent the change in the odds of nocturnal pain associated with a one-unit increase. KL grade was entered as an ordinal covariate; its OR represents the change associated with a one-grade increase. The WOMAC total score was expressed on a 0–100 scale. No problematic multicollinearity was detected (VIF range 1.105–1.783; tolerance range 0.561–0.905). The intercept was not included in the table. B: logistic regression coefficient; SE: standard error; OR: odds ratio; CI: confidence interval; VIF: variance inflation factor; NP: nocturnal pain; BMI: body mass index; KL: Kellgren–Lawrence; WOMAC: Western Ontario and McMaster Universities Osteoarthritis Index.

## Data Availability

The datasets for this study are not publicly available due to patient confidentiality but are available from the corresponding author upon reasonable request.

## References

[1] Litwic A., Edwards M.H., Dennison E.M., Cooper C. (2013). Epidemiology and burden of osteoarthritis. Br. Med. Bull..

[2] Majjad A., Debbarh Z., El Kacem E.M., Bidah S., Bezza A. (2026). Rheumatologists’ Knowledge of Physical Activity in Knee Osteoarthritis: Findings From a Nationwide Survey. Cureus.

[3] Cigdem-Karacay B., Eraslan S.S., Canli İ., Turhan A. (2026). The effectiveness of virtual reality-based exercises in patients with symptomatic knee osteoarthritis: Randomized controlled study. Rev. Assoc. Med. Bras..

[4] Kanu C., Aldhouse N.V.J., Seçinti E., Edge H., Mellor K., Giblin K., Robinson R., Brumm J.F., Knight S.L. (2025). The Patient Experience of Living with Knee Osteoarthritis Pain: Development of a Conceptual Model. Musculoskelet. Care.

[5] Gümürdü G., Koca T.T., Koçyiğit B.F., Berk E., Nacitarhan V., Kurtgil M.E. (2019). The Relationship of Neuropathic Pain and Central Sensitization with Disease Severity and Radiological Staging in Knee Osteoarthritis. J. PMR Sci..

[6] Perrot S. (2015). Osteoarthritis pain. Best Pract. Res. Clin. Rheumatol..

[7] van Berkel A.C., Ringelenberg R., Bindels P.J.E., Bierma-Zeinstra S.M.A., Schiphof D. (2023). Nocturnal pain, is the pain different compared with pain during the day? An exploratory cross-sectional study in patients with hip and knee osteoarthritis. Fam. Pract..

[8] Petersen K.K., Ciampi de Andrade D., Pickering G., Giordano R., Hertel E., Edwards R.R., Pogatzki-Zahn E., Mobasheri A., Arendt-Nielsen L. (2026). The complexity of pain in osteoarthritis. Nat. Rev. Rheumatol..

[9] Salaffi F., Carotti M., Farah S., Ciccullo C., Gigante A.P., Bandinelli F., Di Carlo M. (2025). A Mediation Appraisal of Neuropathic-like Symptoms, Pain Catastrophizing, and Central Sensitization-Related Signs in Adults with Knee Osteoarthritis—A Cross-Sectional Study. J. Pers. Med..

[10] Valdes A.M., Suokas A.K., Doherty S.A., Jenkins W., Doherty M. (2014). History of knee surgery is associated with higher prevalence of neuropathic pain-like symptoms in patients with severe osteoarthritis of the knee. Semin Arthritis Rheum..

[11] Woolhead G., Gooberman-Hill R., Dieppe P., Hawker G. (2010). Night pain in hip and knee osteoarthritis: A focus group study. Arthritis Care Res..

[12] Chen M.L., Du Y.Q., Xu B.Y., Zhao F., Hu X.Q., Wang Y., Zhang Y. (2026). New insights into the pain of knee osteoarthritis: The characteristics of deep pain and abnormal central processing. Sheng Li Xue Bao.

[13] Burnett W.D., Kontulainen S.A., McLennan C.E., Hazel D., Talmo C., Hunter D.J., Wilson D.R., Johnston J.D. (2015). Knee osteoarthritis patients with severe nocturnal pain have altered proximal tibial subchondral bone mineral density. Osteoarthr. Cartil..

[14] Chen Q., Hayman L.L., Shmerling R.H., Bean J.F., Leveille S.G. (2011). Characteristics of chronic pain associated with sleep difficulty in older adults: The Maintenance of Balance, Independent Living, Intellect, and Zest in the Elderly (MOBILIZE) Boston study. J. Am. Geriatr. Soc..

[15] Sasaki E., Ota S., Chiba D., Kimura Y., Sasaki S., Ando M., Yamamoto Y., Tsuda E., Ishibashi Y. (2021). Association Between Central Sensitization and Increasing Prevalence of Nocturnal Knee Pain in the General Population with Osteoarthritis from the Iwaki Cohort Study. J. Pain Res..

[16] Imamura M., Imamura S.T., Kaziyama H.H., Targino R.A., Hsing W.T., de Souza L.P.M., Cutait M.M., Fregni F., Camanho G.L. (2008). Impact of nervous system hyperalgesia on pain, disability, and quality of life in patients with knee osteoarthritis: A controlled analysis. Arthritis Rheum..

[17] Kılıçaslan H.Ö., Genç A., Tuncer S. (2022). Central sensitization in osteoarthritic knee pain: A cross-sectional study. Turk. J. Phys. Med. Rehabil..

[18] International Association for the Study of Pain IASP Terminology. https://www.iasp-pain.org/Education/Content.aspx?.

[19] Sarıyıldız M.A., Batmaz İ., Kaya M.C., Bozkurt M., Okçu M., Yıldız M., Yazmalar L., Çelepkolu T. (2013). Association of the sleep quality with pain, radiological damage, functional status and depressive symptoms in patients with knee osteoarthritis. Int. J. Caring Sci. JCEI.

[20] Gooberman-Hill R., Sansom A., Sanders C.M., Dieppe P.A., Horwood J., Learmonth I.D., Williams S., Donovan J.L. (2010). Unstated factors in orthopaedic decision-making: A qualitative study. BMC Musculoskelet. Disord..

[21] Sasaki E., Tsuda E., Yamamoto Y., Maeda S., Inoue R., Chiba D., Okubo N., Takahashi I., Nakaji S., Ishibashi Y. (2014). Nocturnal knee pain increases with the severity of knee osteoarthritis, disturbing patient sleep quality. Arthritis Care Res..

[22] Alhasan M.K., Obeidat N., Al Bdour I.R., Al Zeghari M.J., Al-Othman L.E. (2025). Radiographic predictors of knee osteoarthritis. Radiography.

[23] Lee N.K., Won S.J., Lee J.Y., Kang S.B., Yoo S.Y., Chang C.B. (2022). Presence of Night Pain, Neuropathic Pain, or Depressive Disorder Does Not Adversely Affect Outcomes After Total Knee Arthroplasty: A Prospective Cohort Study. J. Korean Med. Sci..

[24] Bellamy N., Buchanan W.W., Goldsmith C.H., Campbell J., Stitt L.W. (1988). Validation study of WOMAC: A health status instrument for measuring clinically important patient relevant outcomes to antirheumatic drug therapy in patients with osteoarthritis of the hip or knee. J. Rheumatol..

[25] Tüzün E.H., Eker L., Aytar A., Daşkapan A., Bayramoğlu M. (2005). Acceptability, reliability, validity and responsiveness of the Turkish version of WOMAC osteoarthritis index. Osteoarthr. Cartil..

[26] Kellgren J.H., Lawrence J.S. (1957). Radiological assessment of osteo-arthrosis. Ann. Rheum. Dis..

[27] Bouhassira D., Attal N., Alchaar H., Boureau F., Brochet B., Bruxelle J., Cunin G., Fermanian J., Ginies P., Grun-Overdyking A. (2005). Comparison of pain syndromes associated with nervous or somatic lesions and development of a new neuropathic pain diagnostic questionnaire (DN4). Pain.

[28] Unal-Cevik I., Sarioglu-Ay S., Evcik D. (2010). A comparison of the DN4 and LANSS questionnaires in the assessment of neuropathic pain: Validity and reliability of the Turkish version of DN4. J. Pain.

[29] Neblett R., Cohen H., Choi Y., Hartzell M., Williams M., Mayer T.G., Gatchel R.J. (2013). The Central Sensitization Inventory (CSI): Establishing clinically significant values for identifying central sensitivity syndromes in an outpatient chronic pain sample. J. Pain.

[30] Neblett R., Hartzell M.M., Mayer T.G., Cohen H., Gatchel R.J. (2017). Establishing clinically relevant severity levels for the central sensitization inventory. Pain Pract..

[31] Mayer T.G., Neblett R., Cohen H., Howard K.J., Choi Y.H., Williams M.J., Perez Y., Gatchel R.J. (2012). The development and psychometric validation of the central sensitization inventory. Pain Pract..

[32] Düzce Keleş E., Birtane M., Ekuklu G., Kılınçer C., Çalıyurt O., Taştekin N., Is E.E., Ketenci A., Neblett R. (2021). Validity and reliability of the Turkish version of the central sensitization inventory. Arch. Rheumatol..

[33] Buysse D.J., Reynolds C.F., Monk T.H., Berman S.R., Kupfer D.J. (1989). The Pittsburgh Sleep Quality Index: A new instrument for psychiatric practice and research. Psychiatry Res..

[34] Ağargün M.Y., Kara H., Anlar Ö. (1996). Pittsburgh uyku kalitesi indeksi’nin geçerliği ve güvenirliği. Türk Psikiyatr. Derg..

[35] Craig C.L., Marshall A.L., Sjostrom M., Bauman A.E., Booth M.L., Ainsworth B.E., Pratt M., Ekelund U.L., Yngve A., Sallis J.F. (2003). International physical activity questionnaire: 12-country reliability and validity. Med. Sci. Sports Exerc..

[36] Öztürk M. (2005). A Research on Reliability and Validity of International Physical Activity Questionnaire and Determination of Physical Activity Level in University Students. Master’s Thesis.

[37] Fingleton C., Smart K., Moloney N., Fullen B.M., Doody C. (2015). Pain sensitization in people with knee osteoarthritis: A systematic review and meta-analysis. Osteoarthr. Cartil..

[38] Hochman J.R., Gagliese L., Davis A.M., Hawker G.A. (2011). Neuropathic pain symptoms in a community knee OA cohort. Osteoarthr. Cartil..

[39] Power J.D., Perruccio A.V., Badley E.M. (2005). Pain as a mediator of sleep problems in arthritis and other chronic conditions. Arthritis Rheum..

[40] Kuralay C., Kiyak E. (2018). Sleep Quality and Factors Affecting Patients with Knee Osteoarthritis. Int. J. Caring Sci..

